# I. Phillips

**Published:** 1889-07

**Authors:** 


See new advertisement of Isaac Phillips the surgical instrument man in Atlanta.

